# Clinical Impact of Semaglutide Beyond Glycemic Control: A Critical Analysis of Oncogenic Potential and Mitigation of Cardiotoxicity

**DOI:** 10.3390/ph19020297

**Published:** 2026-02-10

**Authors:** Adriana Correra, Alfredo Mauriello, Valeria Cetoretta, Anna Chiara Maratea, Lucia Riegler, Isabella Di Sarno, Francesco Giallauria, Federico Guerra, Vincenzo Russo, Antonello D’Andrea

**Affiliations:** 1 Cardiology Department, Ospedali Riuniti University Hospital, Viale Pinto 1, 71122 Foggia, Italy; adrianacorrera@gmail.com; 2 S.C. Cardiology, Institute National Cancer, IRCCS, Fondazione “G. Pascale”, Via M. Semmola 52, 80131 Naples, Italy; alfredo.mauriello93@libero.it; 3 Cardiology and Arrhythmology Clinic, Marche Polytechnic University, University Hospital “Ospedali Riuniti”, Via Conca 71, 60126 Ancona, Italy; vcetoretta@gmail.com (V.C.); guerra.fede@gmail.com (F.G.); 4 Department of Cardiovascular Disease, ASL Napoli 1 Centro, Via Comunale del Principe, 13/a, 80145 Naples, Italy; annachiara.maratea@gmail.com; 5 Cardiology and Intensive Care Unit, Department of Cardiology, “Umberto I” Hospital, Via Alfonso De Nicola 1, 84014 Nocera Inferiore, Italy; luciariegler@yahoo.it; 6 UOSD Neurophysiopathology, AORN “Santobono Pausillipon”, Via Mario Fiore, 6, 80129 Naples, Italy; isadisarno@gmail.com; 7 Department of Translational Medical Sciences, “Federico II” University of Naples, Via S. Pansini 5, 80131 Naples, Italy; francesco.giallauria@unina.it; 8 Cardiology Unit, Department of Medical and Translational Sciences, University of Campania “Luigi Vanvitelli”, “V. Monaldi” Hospital, Via Leonardo Bianchi snc, 80131 Naples, Italy; vincenzo.russo@unicampania.it

**Keywords:** semaglutide, GLP-1RA, oncogenesis, cardiotoxicity, cardiovascular diseases, obesity

## Abstract

**Introduction**: Semaglutide, a glucagon-like peptide-1 receptor agonist (GLP-1RA), has demonstrated unprecedented efficacy in the treatment of type 2 diabetes mellitus (T2DM) and obesity. However, its rapid clinical widespread use has ignited a debate regarding long-term safety, particularly concerning the risk of specific neoplasms and its ability to modulate cardiovascular health, not only as primary prevention but also as a potential agent to mitigate cardiotoxicity. **Objectives**: This narrative review aims to analyze the most recent evidence from clinical trials and post-marketing surveillance to evaluate the correlation between semaglutide use and the incidence of cancer, as well as the drug’s efficacy in reducing cardiotoxicity induced by anticancer therapies. **Results and Discussion**: While preclinical rodent models suggested a link to medullary thyroid carcinoma, human epidemiological data remain reassuring, though caution is advised in patients with genetic predisposition. Regarding pancreatic cancer, current meta-analyses do not confirm a significant increase in risk, suggesting that metabolic benefits outweigh potential concerns. **Conclusions**: Semaglutide is confirmed as a therapeutic tool with a highly favorable benefit–risk profile. While oncological monitoring must continue, the drug’s cardioprotective and anti-inflammatory properties open new frontiers not only in metabolic management but also in safeguarding cardiovascular integrity in complex clinical scenarios.

## 1. Introduction

Type 2 diabetes (T2D) is a multifaceted metabolic disorder that necessitates highly individualized therapeutic approaches [1]. Beyond essential lifestyle and dietary modifications, pharmacological intervention is typically required to achieve glycemic targets [1]. Glucagon-like peptide-1 receptor agonists (GLP-1RA) have emerged as a pivotal class of treatment, as they enhance insulin secretion and suppress glucagon release in a glucose-dependent manner [2]. This mechanism ensures effective glycemic control while maintaining a favorable safety profile with a low risk of hypoglycemia [3]. Notably, unlike many traditional antidiabetic agents, GLP-1 receptor agonists facilitate significant weight loss by reducing appetite, limiting energy intake, and modulating food reward perception [4]. Since the U.S. Food and Drug Administration (FDA) approved the injectable and oral formulations of semaglutide in December 2017 and September 2019, respectively, a growing body of research has highlighted its extensive pleiotropic effects [5,6]. Emerging evidence underscores its impact not only on the cardiovascular, gastrointestinal, and renal systems but also on its lesser-known influences on the musculoskeletal and central nervous systems [7]. Furthermore, recent discourse has linked semaglutide to altered risks across various malignancies [8] and potential cardioprotective roles during oncological treatments. As the clinical landscape sees a rise in novel anticancer drugs [9], the challenge of treatment-induced cardiotoxicity has become increasingly prominent. Consequently, this narrative review aims to synthesize the most recent data from clinical trials and post-marketing surveillance to evaluate the correlation between semaglutide use and cancer incidence, while also exploring the drug’s efficacy in mitigating cardiotoxicity associated with anticancer therapies.

## 2. Methods

This paper is a narrative review designed to provide a comprehensive synthesis and critical analysis of current knowledge, recent advancements, and future perspectives regarding the relationship between semaglutide and cancer. Narrative reviews serve as an essential tool for integrating evidence in rapidly evolving multidisciplinary fields, such as cardio-oncology.

To identify relevant literature, we conducted a structured search focused on the core concepts of the study. Following established standards [10] for cardio-oncology reviews, the search was performed using the PubMed/MEDLINE and EMBASE databases, covering the period from January 2015 to January 2026. The search strategy employed Boolean operators (AND, OR) to combine key terms across the following domains:

- Pharmacotherapeutic Agents: ‘Semaglutide’, ‘Glucagon-like peptide-1 (GLP-1)’, and ‘GLP-1 receptor agonists (GLP-1RA)’.
- Clinical Conditions: ‘Cardiotoxicity’, and ‘Cancer’.
- Clinical Assessment: ‘Diagnosis’, ‘Biomarkers’, and ‘Therapeutic management’.

The selection process prioritized English-language articles, including peer-reviewed original research, both retrospective and prospective, comprehensive reviews, and expert editorials [10]. We specifically sought documents providing mechanistic or clinical insights into the interplay between semaglutide, oncogenesis, and its potential role in managing anticancer therapy-induced cardiotoxicity. While this narrative approach does not strictly adhere to the formal replicability of a systematic review, this methodology ensures an evidence-based framework that captures the most significant clinical and scientific developments in the field.

## 3. Semaglutide: Structure and Mechanism

Semaglutide is a long-acting GLP-1RA, a molecule that has radically transformed the therapeutic paradigm for T2DM and obesity [11]. Chemically, semaglutide shares 94% sequence homology with native human GLP-1 but has been engineered with specific structural modifications to overcome the pharmacokinetic limitations of the endogenous hormone [12]. The substitution of alanine with alpha-aminoisobutyric acid at position 8 confers intrinsic resistance to enzymatic degradation by dipeptidyl peptidase-4 (DPP-4), while the attachment of a C18 fatty acid side chain to lysine at position 26 increases its affinity for serum albumin. These modifications result in a prolonged half-life of approximately 168 h, allowing for once-weekly administration and ensuring steady plasma levels that facilitate continuous receptor activation [12].

### 3.1. Molecular Mechanism and Intracellular Signaling

The primary mechanism of action of semaglutide lies in its high-affinity binding to the GLP-1 receptor (GLP-1R), a G-protein-coupled receptor widely expressed not only in the endocrine pancreas but also in the lungs, heart, kidneys, central nervous system, and immune cells [13].

Receptor activation triggers a complex biochemical cascade. The stimulation of the Gαs subunit activates adenylate cyclase, leading to an increase in intracellular levels of cyclic adenosine monophosphate (cAMP) [14]. This increase activates two main pathways, such as Protein Kinase A (PKA) and the exchange protein directly activated by cAMP (EPAC), which together mediate glucose-stimulated insulin exocytosis from pancreatic β-cells [14].

**Figure 1:**
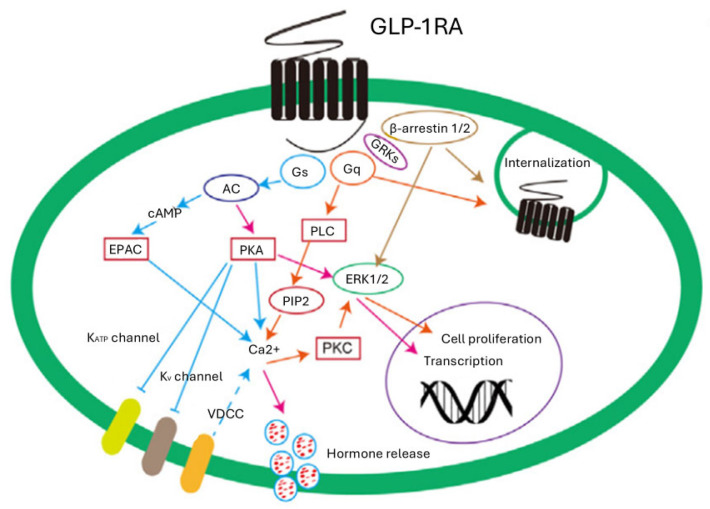
Schematic representation of GLP1R-mediated signaling pathways. AC: adenylate cyclase; cAMP: cyclic adenosine monophosphate; EPAC: exchange protein directly activated; ERK1/2: extracellular signal-regulated kinase; GLP-1RA: glucagon-like peptide-1 receptor agonist; GRKs: glicentin-related kinases; K_ATP_ channel: ATP-sensitive potassium channel; K_V_ channel: voltage-dependent K+ channels; PK: protein kinase; PIP2: phosphatidylinositol 4,5-bisphosphate or PtdIns (4,5)P2; PLC: phospholipase C; VDCC: voltage-dependent Ca2+ channel. Blue: Gs pathway; Red: AC pathway; Orange: Gq pathway; Brown: β-arrestin pathway.

Beyond the cAMP pathway, semaglutide stimulates the phosphatidylinositol 3-kinase (PI3K) and Protein Kinase B (Akt) pathways [15]. This signaling route is important for the drug’s cytoprotective and anti-apoptotic effects; in hepatocytes and muscle cells, Akt activation improves insulin sensitivity and regulates gluconeogenesis. Recent preclinical studies [16,17,18,19,20,21,22,23,24,25,26] have also highlighted that semaglutide acts through the activation of AMP-activated protein kinase (AMPK), improving mitochondrial metabolism and promoting autophagy, which are fundamental processes for cell survival under metabolic or pharmacological stress [27]. Figure 1 is a schematic representation of GLP1R-mediated signaling pathways.

### 3.2. Systemic Effects: Glycemic Control and Weight Management

At the systemic level, the effects of semaglutide manifest through the fine-tuning of glucose homeostasis [28]. The drug enhances glucose-dependent insulin secretion while simultaneously suppressing inappropriate glucagon release from pancreatic α-cells, thereby reducing hepatic glucose production [3]. This dual effect drastically minimizes the risk of hypoglycemia compared to traditional insulin therapies [3].

A hallmark effect of semaglutide is its ability to induce significant weight loss, averaging over 15% of total body weight [29]. In the Semaglutide Effects on Cardiovascular Outcomes in People With Overweight or Obesity (SELECT) trial, enrolling 17,604 adults with preexisting cardiovascular disease, overweight or obesity, without T2D, at 208 weeks, semaglutide was associated with mean reduction in weight (−10.2%), waist circumference (−7.7 cm) and waist-to-height ratio (−6.9%) versus placebo (−1.5%, −1.3 cm and −1.0%, respectively; *p* < 0.0001 for all comparisons versus placebo) [29]. This outcome is mediated by the activation of GLP-1 receptors in the central nervous system, particularly in the hypothalamus and brainstem, which reduces appetite, increases satiety, and modulates food preferences, leading to a spontaneous reduction in caloric intake [30]. Furthermore, semaglutide slows gastric emptying, contributing to the reduction of postprandial glycemic peaks [31].

### 3.3. Cardiovascular and Anti-Atherosclerotic Effects

Semaglutide exerts profound protective effects on the cardiovascular system, as documented in large-scale clinical trials such as SUSTAIN-6 [32] and SELECT [29], which showed a reduction of up to 26% in major adverse cardiovascular events (MACE). The drug acts directly on the vascular endothelium by improving nitric oxide production and reducing vascular inflammation through the downregulation of adhesion molecules such as vascular cell adhesion molecule (VCAM)-1 and metalloproteinases (MMP), such as MMP-3 [33,34,35].

Additionally, semaglutide promotes a metabolic “switch” from white adipose tissue (WAT) to brown adipose tissue (BAT), enhancing thermogenesis and improving lipid profiles, with documented reductions in low-density lipoprotein (LDL) cholesterol and triglycerides. These effects, combined with reduced blood pressure and visceral fat mass, mitigate cardiovascular risk factors independently of glycemic control [36,37,38].

## 4. Risk of Cancers Regarding the Use of Semaglutide

The relationship between the use of semaglutide and the development of cancer is a widely studied topic. Current evidence, based on extensive meta-analyses of randomized controlled trials (RCTs) and real-world studies, indicates that there is no significant increase in oncogenic risk in humans.

Analyses conducted on tens of thousands of patients demonstrate that the incidence of all types of neoplasms (benign, malignant, or unspecified) is similar between groups treated with semaglutide and control groups (placebo or other antidiabetic drugs) [8]. A meta-analysis of 21 RCTs involving 16,839 people either on semaglutide or placebo investigated the impact of semaglutide on the occurrence of cancers. Compared to placebo, occurrence of pancreatic cancer [OR 0.25 (95%CI: 0.03–2.24); *p* = 0.21], thyroid cancer [OR 2.04 (95% CI: 0.33–12.61); *p* = 0.44; I^2^ = 0%] and all neoplasms (benign, malignant and otherwise unspecified) [OR 0.95 (95% CI: 0.62–1.45); *p* = 0.82; I^2^ = 0%] was similar in the semaglutide group [8].

Data from over 1.1 million patients suggest that GLP-1 agonists, particularly semaglutide, may even be associated with a reduced risk for several types of cancer, including gastrointestinal, breast, skin, and prostate cancers [39]. Analysis revealed significant cancer-risk reductions associated with GLP-1RA use across multiple cancer types compared to matched controls. Notable risk reductions were observed in gastrointestinal (HR 0.67, 95% CI 0.59–0.75), skin (HR 0.62, 95% CI 0.55–0.70), breast (HR 0.72, 95% CI 0.64–0.82), female genital (HR 0.61, 95% CI 0.53–0.71), prostate (HR 0.68, 95% CI 0.58–0.80), and lymphoid/hematopoietic cancers (HR 0.69, 95% CI 0.60–0.80). Semaglutide demonstrated superior protective effects, particularly in gastrointestinal cancers (HR 0.45, 95% CI 0.37–0.53). Conversely, liraglutide showed increased risks for thyroid (HR 1.70, 95% CI 1.03–2.82) and respiratory cancers (HR 1.62, 95% CI 1.13–2.32) [40].

### 4.1. Focus on Thyroid Carcinoma

C-cells are much more abundant in the rodent thyroid. GLP-1 receptor agonists stimulated calcitonin release, up-regulation of calcitonin gene expression, and subsequently C-cell hyperplasia in rats and, to a lesser extent, in mice. In contrast, humans and/or cynomolgus monkeys had low GLP-1 receptor expression in thyroid C-cells, and GLP-1 receptor agonists did not activate adenylate cyclase or generate calcitonin release in primates [41]. Although “black box” warnings exist due to rodent studies showing a link between GLP-1RAs and medullary thyroid carcinoma (MTC) [42], human clinical data are reassuring. The incidence of thyroid cancer in patients treated with semaglutide is less than 1% [43]. Therefore, specific meta-analyses involving 14,550 participants, with 7830 receiving semaglutide, were analyzed, with an additional number of 18 studies, and they found no statistically significant increase in thyroid cancer risk (OR: 2.04 vs. placebo, but with confidence intervals including the null; OR 1.19 vs. active controls) [43]. In 2023, the European Medicines Agency (EMA)’s Pharmacovigilance Risk Assessment Committee (PRAC) concluded that there is no evidence of a causal relationship between these drugs and thyroid cancer in humans [44].

It is noteworthy that rodent models differ from humans due to anatomical and physiological variations; for instance, rodents exhibit a significantly higher density of GLP-1R expression in thyroid C-cells compared to humans.

### 4.2. Pancreatic Cancer and Other Cancers

Historically, there were concerns regarding pancreatic cancer, but updated data refutes such a correlation. Levy et al. [40] conducted a large-scale cohort study, including 1,119,363 individuals, to examine the effects of GLP-1RA treatment on cancer risk in individuals with obesity. The incidence of pancreatic cancer was found to be extremely low and not higher than in control groups (HR = 0.328, 95% CI = 0.172–0.625 vs. placebo). Conversely, semaglutide seems to have a protective effect in some cohorts, significantly reducing the risk of digestive tract cancer (HR = 0.476, 95% CI = 0.393–0.576). In summary, semaglutide seems not to be associated with an increased risk of cancer in humans. On the contrary, due to improved metabolic control and potential immunomodulatory mechanisms, it may offer preventive benefits against various forms of cancer.

### 4.3. Immunomodulatory Effects and Tumor Microenvironment Remodeling

Emerging evidence suggests that semaglutide possesses immunomodulatory properties that could slow the progression of certain malignancies, countering historical concerns regarding carcinogenesis [45].

In 4T1 mouse breast cancer models, semaglutide increases the accumulation and maturation of dendritic cells (CD11c+), enhancing antigen presentation to cytotoxic T lymphocytes (CD8+), improving activation of adaptive immunity [45].

The drug reduces the infiltration of regulatory T cells (FoxP3+) and the production of immunosuppressive cytokines like IL-10 in the tumor microenvironment (TME), alleviating cancer immune evasion, through Treg suppression [45].

In pancreatic cancer, semaglutide reduces the expression of the enzyme Prolyl 4-hydroxylase (P4HA1) in cancer-associated fibroblasts (pCAFs). This leads to reduced collagen hydroxylation and stromal fibrosis, making the cancer barrier less dense and favoring T-cell infiltration [45].

In papillary thyroid carcinoma, semaglutide induces macrophage polarization toward the antitumor M1 phenotype via the GLP-1R/PPARG/ACSL1 pathway, inhibiting cancer cell proliferation [11].

The available clinical evidence consistently indicates that semaglutide seems not to be associated with an increased risk of cancer in humans, including thyroid and pancreatic malignancies. Meta-analyses of randomized controlled trials provide a reassuring safety signal, showing no significant differences in the incidence of overall neoplasms or site-specific cancers compared with placebo or active comparators. However, it should be acknowledged that most RCTs were not designed or powered to detect cancer-related endpoints, limiting their ability to identify rare or long-latency malignancies. Consequently, these findings should be interpreted as evidence of no detectable excess risk, rather than definitive proof of oncologic neutrality.

Observational and real-world studies suggesting a reduced incidence of several cancer types among GLP-1 receptor agonist users, particularly with semaglutide, warrant a more cautious interpretation. While these associations are intriguing, they remain vulnerable to residual confounding, including confounding by indication, differences in baseline metabolic risk, weight loss-mediated effects, and healthy-user bias. Therefore, the observed cancer risk reductions cannot be unequivocally attributed to a direct antineoplastic effect of semaglutide. Instead, they may reflect indirect benefits driven by improved insulin sensitivity, reduced chronic inflammation, and sustained weight reduction, all of which are known modifiers of cancer risk.

Similarly, concerns regarding pancreatic cancer have not been substantiated by contemporary large-scale analyses, which consistently demonstrate low absolute incidence rates and no excess risk compared with control populations. Taken together, the current body of evidence supports the oncologic safety of semaglutide while highlighting the need to avoid causal inferences regarding cancer prevention. Future studies specifically designed to address cancer-related outcomes, with adequate follow-up and adjustment for metabolic confounders, will be essential to clarify whether the observed associations reflect true biological effects or indirect consequences of metabolic improvement.

## 5. Mitigation Effect of Semaglutide Regarding Cardiotoxicity

One of the most innovative frontiers concerns the effect of semaglutide in preventing chemotherapy-induced cardiotoxicity, such as that caused by doxorubicin, in a pre-clinical study involving thirty-five rats [46]. In a preclinical study involving six-week-old C57/BL6J mice, it seems that semaglutide can attenuate myocardial damage through BNIP3 reduction [47]. Semaglutide acts via the PI3K/Akt pathway to inhibit the expression of BNIP3 in the myocardium, a protein involved in mitochondrial dysfunction and necrotic cell death [47]. Therefore, semaglutide preserves mitochondrial membrane potential and oxygen consumption rate (OCR), reducing the production of reactive oxygen species (ROS) and preventing the energetic collapse of cardiomyocytes. Semaglutide seems to have an anti-apoptotic effect, as a reduction in damage markers such as troponin and creatine kinase (CK-MB) has been observed, supported by decreased expression of caspase-3 and p53 [47].

Taken together, the available data indicate that the proposed cardioprotective and antitumor-related effects of semaglutide are currently supported exclusively by preclinical evidence. As such, these findings do not justify clinical extrapolation but rather constitute a biological proof of concept suggesting that semaglutide may modulate key molecular and immunometabolic pathways relevant to chemotherapy-induced cardiotoxicity. In particular, the inhibition of BNIP3 expression through activation of the PI3K/Akt pathway represents a mechanistically coherent signal, as BNIP3 is a central mediator of mitochondrial dysfunction, energetic failure, and necrosis-like cardiomyocyte injury. This mechanism suggests that any potential cardioprotective role of semaglutide may be especially relevant in anticancer treatments characterized by prominent mitochondrial damage and myocardial necrosis, such as anthracycline-based regimens.

Beyond direct myocardial effects, semaglutide appears to exert a broader immunomodulatory influence, including remodeling of the tumor microenvironment through enhanced dendritic cell maturation, reduced regulatory T-cell infiltration, and attenuation of immunosuppressive signaling. Although these effects remain context-dependent and incompletely characterized, they provide a plausible framework by which semaglutide could indirectly counteract tumor immune escape mechanisms shared across multiple malignancies. Moreover, preclinical studies suggest that semaglutide may interact with tumor biology through pathway-specific mechanisms, such as modulation of cancer-associated fibroblast activity or macrophage polarization, rather than through direct cytotoxic effects on tumor cells.

Importantly, no clinical studies to date have evaluated semaglutide as a cardioprotective agent in oncology patients, nor is there robust clinical evidence supporting a direct antineoplastic effect. Therefore, these observations should be interpreted as hypothesis-generating, underscoring the need for dedicated translational and clinical investigations to determine whether semaglutide’s metabolic, mitochondrial, and immunomodulatory actions can be leveraged to prevent cardiotoxicity without compromising oncologic efficacy.

## 6. Future Perspectives

Looking toward future perspectives, current evidence suggests that semaglutide’s profile may evolve from a purely metabolic drug to a foundational pillar in the multidisciplinary management of oncology patients. However, despite encouraging results from meta-analyses and retrospective studies, it is imperative to conduct specific and prospective RCTs to confirm these long-term benefits. These studies must focus not only on rigorous oncological surveillance, especially in individuals with genetic predispositions, but also, and more importantly, on semaglutide’s potential protective role against cancer therapy-induced cardiotoxicity. Only through standardized trials will it be possible to define precise clinical recommendations, evaluate the drug’s interaction with the efficacy of anticancer therapies, and determine whether its anti-inflammatory and cardioprotective properties can truly prevent myocardial damage in fragile populations. The transition toward a “metabolic cardio-oncology” therefore requires high-grade evidence to integrate this GLP-1 agonist into standard care protocols.

## 7. Conclusions

Semaglutide is confirmed as a therapeutic tool with a highly favorable benefit–risk profile. While initial preclinical rodent models suggested a link to MTC, human epidemiological data remain reassuring, as they do not confirm a significant increase in oncogenic risk, even regarding pancreatic cancer. Conversely, the drug’s cardioprotective and anti-inflammatory properties open new clinical frontiers, suggesting a potential role in safeguarding cardiovascular integrity even in complex scenarios, such as the mitigation of cardiotoxicity induced by anticancer therapies. Despite the necessity to maintain rigorous oncological monitoring, current evidence indicates that the metabolic and systemic benefits of semaglutide outweigh potential concerns, positioning it as a key resource in the modern multidisciplinary management of the patient.

## Data Availability

No new data were created or analyzed in this study. Data sharing is not applicable to this article.
